# Xanthan Gum as an Adjuvant in a Subunit Vaccine Preparation against Leptospirosis

**DOI:** 10.1155/2014/636491

**Published:** 2014-05-07

**Authors:** Katia L. Bacelo, Daiane D. Hartwig, Fabiana K. Seixas, Rodrigo Schuch, Angelita da S. Moreira, Marta Amaral, Tiago Collares, Claire T. Vendrusculo, Alan J. A. McBride, Odir A. Dellagostin

**Affiliations:** Unidade de Biotecnologia, Centro de Desenvolvimento Tecnológico, Universidade Federal de Pelotas, 96010-900 Pelotas, RS, Brazil

## Abstract

Leptospiral immunoglobulin-like (Lig) proteins are of great interest due to their ability to act as mediators of pathogenesis, serodiagnostic antigens, and immunogens. Purified recombinant LigA protein is the most promising subunit vaccine candidate against leptospirosis reported to date, however, as purified proteins are weak immunogens the use of a potent adjuvant is essential for the success of LigA as a subunit vaccine. In the present study, we compared xanthan pv. pruni (strain 106), aluminium hydroxide (alhydrogel), and CpG ODN as adjuvants in a LigA subunit vaccine preparation. Xanthan gum is a high molecular weight extracellular polysaccharide produced by fermentation of *Xanthomonas* spp., a plant-pathogenic bacterium genus. Preparations containing xanthan induced a strong antibody response comparable to that observed when alhydrogel was used. Upon challenge with a virulent strain of *L. interrogans* serovar Copenhageni, significant protection (Fisher test, *P* < 0.05) was observed in 100%, 100%, and 67% of hamsters immunized with rLigANI-xanthan, LigA-CpG-xanthan, and rLigANI-alhydrogel, respectively. Furthermore, xanthan did not cause cytotoxicity in Chinese hamster ovary (CHO) cells *in vitro*. The use of xanthan as an adjuvant is a novel alternative for enhancing the immunogenicity of vaccines against leptospirosis and possibly against other pathogens.

## 1. Introduction

Leptospirosis is a zoonotic disease that occurs worldwide and is caused by a pathogenic species of* Leptospira*. With 500,000 cases reported each year, this disease remains a significant public health concern [1]. Parenteral immunizations with whole-cell, heat-killed vaccine preparations are very protective, but their use is limited due to severe side effects (pain, nausea, and fever), serovar-restricted protection, and short-term immunity [2]. Recombinant vaccines have the potential to overcome these limitations. Subunit vaccines consist of purified antigens that are specifically recognized by cells of the adaptive immune system. However, they lack intrinsic pathogen-associated molecular patterns (PAMPs) and the ability to activate the immune system in an appropriate way is impaired, so the use of adjuvants is required [3]. Several leptospiral antigens have been evaluated, as established in a recent review by Odir et al., 2011 [4], and recombinant LigA peptides, including the nonidentical carboxy-terminus named LigANI, have been expressed and evaluated in experimental models of leptospirosis using different adjuvants [4–6] and as a DNA vaccine [7], with promising results.

The main adjuvants currently approved for human use by the US Food and Drug Administration (FDA) are aluminium based mineral salts, the most commonly used of which is aluminium hydroxide (generically known as alhydrogel). For decades, alhydrogel has been used successfully in vaccine preparations and has a good safety record [8]. However, alhydrogel is a weak adjuvant for antibody induction against protein subunits and is a poor adjuvant for cell-mediated immunity. In addition, alhydrogel can cause severe local reactions such as erythema, subcutaneous nodules, and contact hypersensitivity [9]. Therefore, there is a need for the development of new adjuvant strategies.

Xanthan is a polysaccharide derived from* Xanthomonas* spp., a plant-pathogenic bacterium genus, which has viscous properties and is widely used as a thickener or viscosifier and a stabilizer in the food industry, as well as other industries [10–12]. Chemically, it is considered an anionic polyelectrolyte, with a cellulosic backbone chain linked to a trisaccharide side chain consisting of two D-mannose units with alternating D-glucuronic acid residues that can be acetylated or pyruvated at different levels, which influences both the chemical and physical properties of xanthan [13]. The intrinsic adjuvant properties of xanthan gum as a murine lymphocyte activator were originally described in the 1980s but have remained largely unexplored in the following decades [14]. More recently, xanthan has been identified in antitumor effects of [13] and it has been successfully used in bioadhesive formulations for intranasal influenza virus immunizations [12, 15].

In the present study, we demonstrated that the rLigANI protein used in combination with xanthan induced protection against lethal challenge in the standard Golden Syrian hamster model for leptospirosis. Together, LigANI and xanthan induced a strong IgG response and the xanthan polysaccharides did not demonstrate cytotoxicity in Chinese hamster ovary (CHO) cells* in vitro*.

## 2. Materials and Methods

### 2.1. Bacterial Strains and Growth Conditions

*L. interrogans* serovar Copenhageni strain FIOCRUZ L1-130 was cultivated at 30°C in Ellinghausen-McCullough-Johnson-Harris (EMJH) liquid medium, supplemented with Leptospira Enrichment EMJH (Difco, USA). Bacterial growth was monitored using dark-field microscopy.* Escherichia coli* strain BL21 (DE3) pLysS (Invitrogen) was grown in Luria-Bertani (LB) medium (1% tryptone, 0.5% yeast extract, 0.5% NaCl, and 2% agar) at 37°C with the addition of 50 *μ*g·mL^−1^ chloramphenicol and 100 *μ*g·mL^−1^ ampicillin.

### 2.2. Ethics Statement

Animal experiments described in this study were carried out in strict accordance with the guidelines of the National Council for Control of Animal Experimentation, Brazil (CONCEA, n° 11,794), and approved by the Ethics Committee in Animal Experimentation, Federal University of Pelotas, Brazil (Permit number: 7777). Hamsters were monitored daily and were euthanized upon the appearance of clinical symptoms of leptospirosis. All surgery was performed under sodium pentobarbital anesthesia, and all efforts were made to minimize suffering.

### 2.3. Xanthan Production

Xanthan gum used in this study was produced by* X. arboricola* pv. pruni strain 106 in a 10 L bioreactor (BioStat B Braun Biotech International) with 7 L of fermentation medium, as previously described [16]. The fermented broth was heated to 121°C for 15 min, and the polysaccharides were recovered by precipitation with 96% ethanol, dried at 56°C until maintaining a constant weight, and then powdered to particle size using 60–150 mesh. The xanthan pruni used in these experiments was pooled from four fermentations and characterized by viscosity, moisture, ash nitrogen, acetyl, and pyruvate content, as previously described by Burdock [17] and the Food and Agriculture Organization of the United Nations (FAO) [18].The quantification of the monosaccharides and derivative acids was determined as previously described [19].

### 2.4. rLigANI Subunit Vaccine Preparation

The cloning, expression, and purification of the rLigANI polypeptide were performed as previously described [20];the* Escherichia coli* strain BL21 (DE3) pLysS was used for recombinant protein expression. Purified rLigANI was used in a subunit vaccine preparation with one of three adjuvants: xanthan, alhydrogel, or CpG ODN. Xanthan was diluted with purified water (1.25%, w/v) and stirred until uniformly distributed. The xanthan solution was added to a final concentration of 0.5% (w/v) [15]. When alhydrogel (Bio-Manguinhos/Fiocruz) was used in the vaccine preparation it was added to a final concentration of 15% [21]. The vaccine preparation that contained CpG ODN was comprised of 10 *μ*g phosphotioated CpG ODN (5′-TCG TCG TCG TTC GAA CGA CGT TGA T) also known as ODN-M362 (Alpha DNA, Canada) as described previously [22].

The antigenicity of vaccine preparations containing rLigANI were evaluated by Western Blotting (WB). The rLigANI vaccines were electrotransferred to a nitrocellulose membrane (Hybond ECL, GE Healthcare) and, after incubation in blocking buffer (0.05 M PBS pH 7.4, 0.05% (v/v) tween 20, 5% (PBS-T) and (w/v) nonfat dried milk) overnight at 4°C, the membranes were subjected to three washes (5 min per wash) in PBS-T and incubated for 1 h with mouse polyclonal anti-LigAni antibody (1 : 300 in PBS) followed by 3 washes (5 min per wash) in PBS-T. Rabbit anti-mouse IgG peroxidase conjugate (Sigma Aldrich), diluted 1 : 6,000 in PBS, was added and incubated for 1 h at 37°C. The membranes were washed (5x in PBS-T), and the reaction was developed using 3,3-diamino-benzide-tetra-hydrochloride (DAB) (Sigma Aldrich).

### 2.5. Xanthan* In Vitro* Cytotoxicity

The viability of CHO cells was determined by measuring the reduction of soluble MTT [3-(4,5-dimethylthiazol-2-yl)-2,5-diphenyltetrazolium bromide] compared with water insoluble formazan [12]. Briefly, cells were seeded at a density of 2 × 10^4^ cells per well in a volume of 100 *μ*L in 96-well plates and grown at 37°C in a humidified atmosphere of 5% CO_2_ for 24 h prior to the cell viability assay. The CHO cells were incubated with different concentrations of aqueous xanthan solution (0.25, 0.5, and 1.0% w/v) for 24 h. The media was removed and 180 *μ*L of medium and 20 *μ*L of MTT (5 mg MTT/mL solution) were added to each well. The plates were incubated for an additional 3 h, and the medium was discarded. Two hundred microliters of DMSO was added to each well, and the formazan was solubilized by shaking for 5 min at 100 × g. The absorbance of each well was read on a microplate reader (MR-96A, Mindray Shenzhen, China) at a wavelength of 492 nm. The cell inhibitory growth rate (%) was determined as follows: inhibitory rate = (1 − Abs_492  treated  cells_/Abs_492  control  cells_) × 100. All observations were validated by at least three independent experiments performed in triplicate.

The rate of apoptosis was determined using the Guava Nexin assay (Guava Technologies), according to the manufacturer's instructions. Cells were treated with aqueous xanthan at 0.25, 0.5, and 1.0% for 48 h. Briefly, 2.0 × 10^4^ to 1.0 ×10^5^ cells (100 *μ*L) were added to 100 *μ*L of Guava Nexin Reagent. The cells were incubated in the dark at room temperature for 20 min and samples (2,000 cells per well) were analysed using the flow cytometer Guava EasyCyte System. In this assay, an annexin V-negative and 7-AAD-positive results indicated nuclear debris, annexin V-positive and 7-AAD-positive indicated late apoptotic cells, annexin V-negative and 7-AAD-negative indicated live healthy cells, and annexin V-positive and 7-AAD-negative indicated early apoptotic cells.

### 2.6. Hamster Immunization

Female golden Syrian hamsters 5-6 weeks old, weighing 82.13 g ± 5.39, were divided into nine groups consisting of 6 animals each: group 1: 15% alhydrogel in PBS (alhydrogel-PBS); group 2: rLigANI in PBS (rLigANI); group 3: rLigANI in 15% alhydrogel (rLigANI-alhydrogel); group 4: CpG in PBS (CpG-PBS); group 5: rLigANI and CpG (rLigANI-CpG); group 6: xanthan in PBS (xanthan-PBS); group 7: rLigANI and xanthan (rLigANI-xanthan); group 8: rLigANI, CpG, and xanthan (rLigANI-CpG-xanthan); group 9: bacterin vaccine consisting of 1 × 10^9^ heat-killed whole-leptospires (bacterin), produced as previously described [23]. Two independent experiments were carried out using 50 *μ*g of recombinant protein, with a standard volume of 500 *μ*L applied at a single injection site. The animals were immunized subcutaneously on day 0 and boosted on day 14. Blood was collected by retroorbital bleeding from the venous plexus before each immunization and challenge (days 0, 14, and 28); the sera were collected and stored at −20°C.

### 2.7. Hamster Challenge Study

On day 28 after the first immunization, the hamsters were challenged with an intraperitoneal inoculum of 1.3 × 10^3^ leptospiras, equivalent to 36 × the 50% lethal dose (LD_50_) of* L. interrogans* serovar Copenhageni (strain Fiocruz L1-130) [24]. Hamsters were monitored daily and euthanized when clinical signs of terminal disease were observed. Surviving hamsters were euthanized on day 36 after challenge and blood samples were collected by cardiac puncture. Kidney and lung tissues were harvested for culture isolation and histopathology studies as described previously [23]. Two independent challenge experiments were carried out.

### 2.8. Evaluation of the Antibody Response in Immunized Hamsters

Serum samples collected on days 0, 14, and 28 were evaluated for the presence of specific immunoglobulin G (IgG) by an ELISA using rLigANI as the antigen. A checkerboard analysis was performed to identify the optimal antigen concentration and dilutions of the hamster sera and the antibody conjugate. The ELISA plates (Polysorp Surface, Nunc) were coated with 200 ng of rLigANI protein per well, diluted in carbonate-bicarbonate buffer pH 9.6, and incubated overnight. The plates were washed three times with PBS-T and incubated with 200 *μ*L of 5% blocking buffer at 37°C for 1 h. After 3 washes with PBS-T, hamster serum (diluted 1 : 50) was added and the plates were incubated for 1 h at 37°C. After three washes with PBS-T, the goat anti-hamster IgG peroxidase conjugate (Serotec, USA) diluted 1 : 6,000 was added and the plates were incubated at 37°C for 1 h. After 5 PBS-T washes, the reactions were developed using o-phenylenediamine dihydrochloride (Sigma) and hydrogen peroxide. The reaction was stopped by adding 0.1 M sulphuric acid and the absorbance was determined at 492 nm with a microplate reader (MultiskanMCC/340, Titertek Instruments). The mean values were calculated from serum samples that were assayed in triplicate.

### 2.9. Statistical Analysis

The results are expressed as the mean ± SEM and the significant differences between groups were determined using an analysis of variance (ANOVA), *P* values <0.05 were considered statistically significant. Protection against mortality was evaluated using the Fisher exact test using Epi Info 6.04d software (Centers for Disease Control and Prevention (CDC), Atlanta, GA, USA) and the survival curves were compared using a Log-rank analysis (Mantel Cox test) using Prism 5 software (GraphPad Software Inc., La Jolla, CA, USA).

## 3. Results

### 3.1. rLigAni Vaccines Preparations Antigenicity

**Figure 1:**
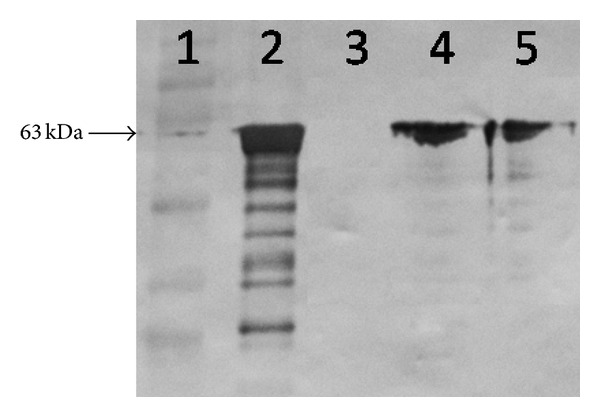
The antigenicity of rLigAni after adjuvant association. The immunoreactivity of rLigAni after association with either CpG or xanthan pruni adjuvants was determined by WB using monoclonal rabbit anti-LigA. Lane 1 has the Full-Range Rainbow Molecular Weight Marker (kDa); Lane 2 shows the purified rLigAni, which is used as a control; Lane 3 contains xanthan-PBS; Lane 4 has rLigAni-xanthan, and Lane 5 shows rLigAni-CpG-xanthan.

The antigenicity of rLigANI associated to xanthan polymer and CpG was evaluated by WB using mouse polyclonal anti-LigAni that recognized a 63 kDa protein in the rLigANI vaccine formulations. The antibody was used to show protein associations and indicated that no significant changes occurred (Figure 1).

### 3.2. Xanthan Characterization

**Figure 2:**
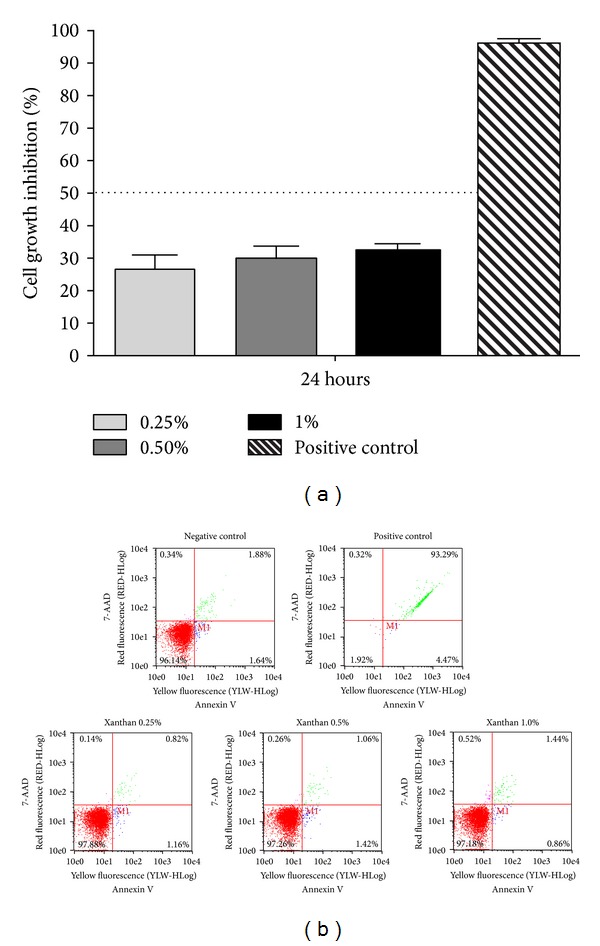
Cytotoxicity of the aqueous xanthan solution. (a) The effect of different concentrations of aqueous xanthan solutions on the inhibition of CHO cells was determined using an MTT assay. The inhibition rate was related to the negative control (DMEM+FBS). Cells were treated with sodium hypochlorite 2% as positive control. The data are expressed as the means ± SEM. (b) After 48 h, the results from the apoptosis assay are shown after flow cytometry and annexin V-PE/7-AAD staining of CHO cells treated with 0.25, 0.5, and 1.0% xanthan solutions and the control groups. The viable cells are in the lower left quadrant, the early apoptotic cells are in the lower right quadrant, the late apoptotic cells, are in the upper right quadrant and the nuclear debris is shown in the upper left quadrant. The numbers indicate the percentage of cells in each quadrant.

The xanthan gum used in this experiment had good viscosity, in accordance with the recommendations by the FAO [18] and Burdock [17] for xanthans used as food additives. The moisture, ash, nitrogen, acetyl, and pyruvate content (Table 1) were in accordance with the recommendations of the FAO [18]. In addition, none of the aqueous xanthan solutions (0.25, 0.5, and 1.0% w/v) demonstrated significant* in vitro* cytotoxicity (Figure 2(a)) and no statistically significant differences in the growth rate were observed at the different xanthan concentrations (*P* > 0.05). Furthermore, the annexin-PE results indicated that the aqueous xanthan solutions did not induce apoptosis at the concentrations tested. The percentage of apoptosis was 1.98, 2.48, and 2.30% when 0.25, 0.5, and 1.0% of aqueous xanthan solutions were used, respectively, which was similar to that observed for the negative control (3.52%) (Figure 2(b)).

### 3.3. Antibody Responses Induced by rLigANI Vaccines

**Figure 3:**
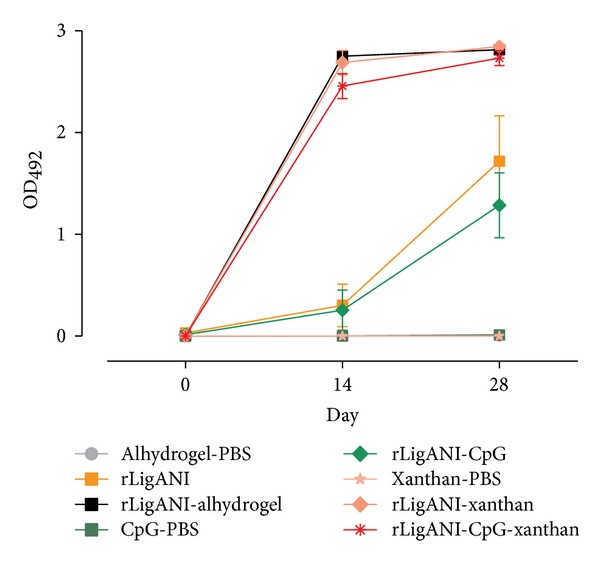
Inducing the humoral immune response in hamsters immunized with different immunogens. Fifty micrograms of the recombinant protein were used. The groups of hamsters were immunized subcutaneously on day 0 and boosted after 2 weeks (day 14). Blood was collected on days 0, 14, and 28 after immunization. The specific IgG responses stimulated by the different immunogens were determined by an ELISA of the hamster serum diluted 1 : 50. The values presented are the means ± SEM for two independent experiments.

Blood samples from hamsters immunized with either alhydrogel-PBS, rLigANI, rLigANI-alhydrogel, CpG-PBS, rLigANI-CpG, xanthan-PBS, rLigANI-xanthan, rLigANI-CpG-xanthan, or the bacterin were collected on days 0, 14, and 28, and the corresponding antibody response was determined by ELISA. After the first immunization, sera from the hamsters immunized with rLigANI-alhydrogel, rLigANI-xanthan, or rLigANI-CpG-xanthan demonstrated significant antibody titres, Figure 3. However, after the boost immunization, all of the hamsters immunized with rLigANI (with or without adjuvant) presented with detectable antibody titres. The bacterin and negative control groups failed to induce detectable levels of IgG antibodies against rLigANI, Figure 3. Although there were localized reactions at the sites of injection that were associated with erythema and alopecia, the hamsters showed no signs of pain or discomfort. No lesions were observed 28 days after immunization. Of note, the* in vitro* tests found that the xanthan was not cytotoxic.

### 3.4. Protective Effect of the Vaccine Preparations following Lethal Challenge with* L. interrogans* Serovar Copenhageni

**Figure 4:**
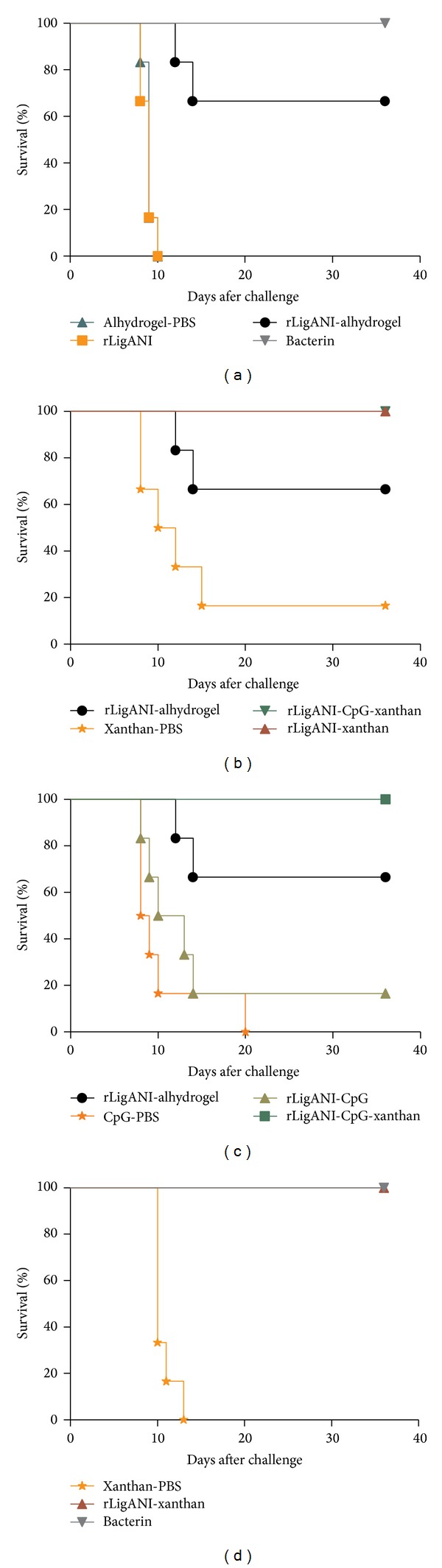
The protective effect of immunization against lethal challenge in a hamster model. Nine- to ten-week-old hamsters were challenged with an intraperitoneal inoculum of 1.3 × 10^3^ leptospires 14 days after the second immunization (day 28). ((a)–(c)) Experiment number 1. (d) Experiment number 2. The survival conferred by rLigANI-alhydrogel against the lethal challenge was statistically significant (*P* < 0.05). The same phenomenon occurred using rLigANI-xanthan and rLigANI-CpG-xanthan as immunogens. The protective effect of immunization using rLigANI-CpG was not significantly different from the negative control group (*P* > 0.05). Fifty micrograms of rLigANI, 0.5% xanthan (w/v), and 10 *μ*g CpG were used. Survival curves were compared using log-rank analysis (Mantel Cox test). Bacterin: heat-killed whole-leptospires.

In the first experiment, hamsters at day 28 after immunization, with an average weight of 128.72 g ± 1.69, were challenged and observed daily for signs of disease. The groups of hamsters immunized with either rLigANI-xanthan, rLigANI-CpG-xanthan, or the bacterin preparation survived (100%), see Table 2 and Figure 4(b). Furthermore, of the hamsters immunized with rLigANI-alhydrogel, compared to the negative alum-PBS control group, a significant number survived (66.7%, Fisher, *P* < 0.05), see Figure 4(a). The same protective effect was achieved in previous study [24]. However, hamsters immunized with rLigANI-CpG were not significantly protected against lethal challenge (16.7% survival) (Figure 4(c)). None of the hamsters in the control groups, alhydrogel-PBS or CpG-PBS, survived and there was only one survivor in the xanthan-PBS group. In addition, rLigANI when administered without an adjuvant failed to protect the hamsters against challenge. In the follow-up challenge experiment to further evaluate the efficacy of the rLigANI-xanthan vaccine preparation, 100% of the hamsters survived challenge and there were no survivors in the control group (Figure 4(d)). The bacterin control group in both experiments conferred 100% protection, Table 2.

Of note, we observed a correlation between the antibody titre and survival in hamsters immunized with the rLigANI-adjuvants preparations (Spearman correlation coefficient 0.6845, *P* < 0.05). Furthermore, the surviving hamster in the rLigANI-CpG group showed significant seroconversion after the first immunization. However, there were no differences between the hamsters that survived and those that did not survive in the rLigANI-alhydrogel group (*t*-test *P* > 0.05).

### 3.5. Histopathology and Culture Isolation

**Figure 5:**
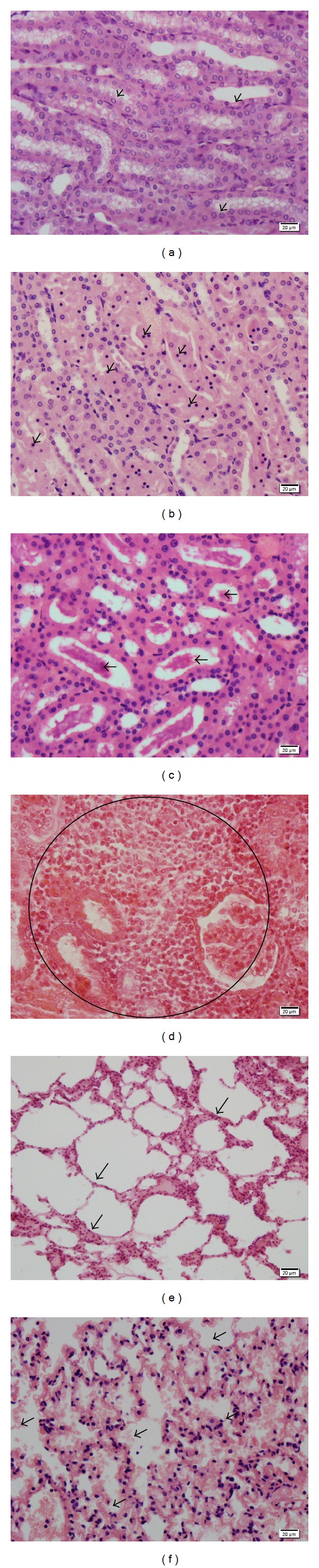
Histopathological changes in organs of hamsters after leptospiral challenge. Panel showing representative HE-stained (400x) kidney ((a)–(d)) and pulmonary ((e) and (f)) sections. (a) Normal tubular kidney epithelium, (b) discrete cell degeneration and necrosis, (c) hyaline deposition, and (d) severe leukocyte infiltration. (e) Normal lung epithelium and (f) moderate oedema and alveolar haemorrhage.

The culture assay showed that 100% (Exp. number 1) and 66.7% (Exp. number 2) of the rLigANI-xanthan and 100% of rLigANI-alhydrogel (Exp. number 1) surviving immunized hamsters harboured leptospires in their kidneys indicating that none of the subunit vaccine preparations afforded sterilizing immunity. None of the groups vaccinated with the bacterin vaccine, showed positive culture (Table 2). The histopathological analyses revealed that the surviving hamsters developed an acute leptospirosis with lesions dominated by moderate pulmonary injury as evidenced by oedema and alveolar haemorrhage. The lesions in the kidneys were characterized by cell degeneration, necrosis, and hyaline deposition (Figure 5). In addition, there were histopathological changes in 33.3% (Exp. number 1) and 16.7% (Exp. number 2) of the kidneys of hamsters in the rLigANI-xanthan groups, and 75% of the rLigANI-alhydrogel group also showed changes in the kidneys. Of note, no kidney changes were observed in the bacterin control group (Table 2).

## 4. Discussion

Using the recombinant protein LigANI as a target for a leptospirosis vaccine yielded promising results, and the best protective effect was obtained using Freund's complete adjuvant [5, 20], an efficient Th1 inducer. However, this caused a high and generally unacceptable level of adverse local effects. In this study, we compared the efficacy of three different adjuvants used in conjunction with LigANI: xanthan polysaccharide, CpG ODN, and alhydrogel. We believe that this is the first study of the application of xanthan as an adjuvant in a recombinant subunit vaccine preparation. In combination with rLigANI, xanthan protected 100% of immunized hamsters (Figure 4). Xanthan has a backbone chain consisting of (1,4) *β*-D-glucan cellulose and is a negatively charged polymer with intrinsic adjuvanticity [14]. It has been used as an FDA-approved food additive and rheology modifier since 1969 and is commonly used as a food thickening agent and stabilizer, demonstrating its biosafety [10, 11]. However, the biological properties and the mechanism of xanthan adjuvanticity are not clear and have remained unexplored until recently.

A previous study found that the oral administration of xanthan gum as a biological response modifier enhances antitumor activity in mice through toll-like receptor (TLR)-4 recognition [13]. This innate immune response is characterized by the production of proinflammatory cytokines, via transcription factor NF-*κ*B. The induction of adaptive immunity through the activation of innate immunity is vital for vaccine development. This pathway leads to the expression of costimulatory molecules that are essential for the induction of an effective adaptive immune response. Studies in mice have demonstrated that signalling through TLRs is sufficient to initiate an adaptive immune response, which is characterized by Th1 induction and antibody production [25, 26]. Although the LPS content was not determined, the composition of commercial and pruni xanthan was previously studied by our group by comparative thin layer chromatography [19, 27]. With the exception of rhamnose, none of carbohydrate components of* Xanthomonas* LPS [28] were detected. Therefore, the antigenicity of xanthan is unlikely to be related to LPS present in the polysaccharide. The rLigANI-xanthan vaccine preparation induced a robust humoral response comparable to the response of the rLigANI-alhydrogel preparation. The antibody-specific titre induced by the rLigANI-CpG ODN preparation was significantly lower than that induced by the xanthan or alhydrogel adjuvants together with rLigANI (Figure 3). It is known that the adjuvant properties of CpG ODNs were markedly improved by ensuring that the ODNs remained in close proximity to the antigen. Physically binding the ODN to the protein, crosslinking the two with alum, or coincorporating them in lipid emulsions or vesicles generated specific IgG responses greater than with antigen alone [29–31]. We questioned if xanthan gum, based on their physicochemical characteristics, could promote this closeness. However, CpG ODN M362 and xanthan coincorporated with rLigANI antigen induced an antibody titre comparable to that produced by the protein and polysaccharide alone. Preclinical studies indicate that CpG ODN improve the activity of vaccines targeting cancer and infectious diseases caused by viruses, bacteria, and protozoa [29, 32]. CpG ODN M362 is a type C human/murine TLR-9 ligand and was used in the present study due to the lack of a specific ligand for a hamster model. Besides the fact that xanthan could not have afforded the proximity between antigen-ODN or the absolute lack of substance for this purpose, as in the case of rLigANI-CpG group, it is possible that ODN M362 is not appropriate for the model studied and studies with other CpG ODNs and associated to alum will be necessary before we can dismiss the use of this adjuvant in subunit vaccines preparations against leptospirosis.

Studies carried out using recombinant LigA polypeptides and alhydrogel as an adjuvant report between 50 [33] and 100% [34] survival; however, in some studies the unvaccinated control group survival rates were over 50%. In the present study, we found a significant protective effect in 4/6 hamsters when alhydrogel was used as the adjuvant. Although the rLigANI-xanthan vaccine preparation protected a greater number of hamsters, 6/6 in two separate challenge experiments, the increased efficacy was not significant compared to rLigANI-alhydrogel. There was a survivor in the control group immunized with xanthan-PBS in the first experiment and while 100% of the hamsters survived, this reduced vaccine efficacy to 83.3%. Xanthan did not show* in vitro* toxicity in CHO cells at the levels tested in the present study. The* in vitro* cytotoxicity of xanthan in chicken splenic macrophages [12] and in L929 mouse fibroblast cells [35] was previously evaluated, and no change in cell viability was observed. Chellat and coworkers [36] investigated the* in vitro* and* in vivo* biocompatibility of a chitosan-xanthan polyionic complex, and they did not observe any cytotoxic effects in male Wistar rats.

## 5. Conclusion

In summary, this study demonstrated the adjuvant effect of xanthan when used with rLigANI, a poorly immunogenic antigen, in a subunit vaccine preparation against leptospirosis. Furthermore, the xanthan polysaccharide enhanced the immune response and the immune protection induced by rLigANI.

## Figures and Tables

**Table 1:** Moisture, ash, nitrogen, acetyl, and pyruvate content (%w/v) of xanthan produced by *X. arboricola* strain 106.

Analysis	Content	Limits (%)*
Moisture	5.0 ± 0.03	≤15
Ash	14.37 ± 0.04	≤16
Nitrogen	1.06 ± 0.01	≤1.5
Acetyl	2.45 ± 0.10	—
Pyruvate	1.93 ± 0.06	—

Values are the means ± SD. *Limits established by FAO, 1999.

**Table 2:** Protection conferred by immunization and culture isolation and histology among survivors.

Vaccine preparation	Exp. number	% Protection (number/total)	% Culture positive	Evidence of lesions (%)		
				Alveolar haemorrhage	Cell degeneration	Leukocyte infiltration
rLigANI	1	0 (0/6)	NA	NA	NA	NA
rLigANI-Al	1	66.7 (4/6)*	100 (4/4)	100 (4/4)	75.0 (3/4)	100 (4/4)
rLigANI-CpG	1	16.7 (1/6)	100 (1/1)	100 (1/1)	100 (1/1)	100 (1/1)
rLigANI-Xa	1	100 (6/6)*	100 (6/6)	100 (6/6)	33.3 (2/6)	100 (6/6)
	2	100 (6/6)*	66.7 (4/6)	100 (6/6)	16.7 (1/6)	33.3 (2/6)
rLigANI-CpG-Xa	1	100 (6/6)*	100 (6/6)	100 (6/6)	33.3 (2/6)	100 (6/6)
Bacterin	1	100 (6/6)*	0 (0/6)	100 (6/6)	0 (0/6)	0 (0/6)
	2	100 (6/6)*	0 (0/6)	100 (6/6)	0 (0/6)	0 (0/6)
Xanthan-PBS	1	16.7 (1/6)	100 (1/1)	100 (1/1)	0 (0/1)	100 (1/1)
	2	0 (0/6)	NA	NA	NA	NA
Al-PBS	1	0 (0/6)	NA	NA	NA	NA
CpG-PBS	1	0 (0/6)	NA	NA	NA	NA

ND: not determined; NA: not applicable; Al: Alhydrogel; Xa: Xanthan.

*Statistically significant compared to the relevant control, Fishers exact test, *P* < 0.05.
